# Is physical exercise and dietary therapy a feasible alternative to cognitive behavior therapy in treatment of eating disorders? A randomized controlled trial of two group therapies

**DOI:** 10.1002/eat.23228

**Published:** 2020-01-16

**Authors:** Therese Fostervold Mathisen, Jan H. Rosenvinge, Oddgeir Friborg, KariAnne Vrabel, Solfrid Bratland‐Sanda, Gunn Pettersen, Jorunn Sundgot‐Borgen

**Affiliations:** ^1^ Department of Sports Medicine Norwegian School of Sport Sciences Oslo Norway; ^2^ UiT—The Arctic University of Norway, Department of Psychology Faculty of Health Sciences Tromsø Norway; ^3^ Research Institute of Modum Bad Vikersund Norway; ^4^ Department of Outdoor Studies, Sports and Physical Education University College of Southeast Norway Bø Norway; ^5^ Department of Health and Caring Sciences, Faculty of Health Sciences UiT—The Arctic University of Norway Tromsø Norway

**Keywords:** affect regulation, binge‐eating disorder, bulimia nervosa, dietary therapy, exercise therapy, physical activity, self‐regulation

## Abstract

**Objective:**

To compare effects of physical exercise and dietary therapy (PED‐t) to cognitive behavioral therapy (CBT) in treatment of bulimia nervosa (BN) and binge‐eating disorder (BED).

**Method:**

The active sample (18–40 years of age) consisted of 76 women in the PED‐t condition and 73 in the CBT condition. Participants who chose not to initiate treatment immediately (*n* = 23) were put on a waiting list. Outcome measures were the eating disorder examination questionnaire (EDE‐Q), Clinical Impairment Assessment (CIA), Satisfaction with Life Scale (SWLS), Beck Depression Inventory (BDI), and numbers in remission at posttreatment, and at 6‐, 12‐, and 24‐months follow‐up.

**Results:**

Both treatment conditions produced medium to strong significant improvements on all outcomes with long‐term effect. The PED‐t produced a faster improvement in EDE‐Q and CIA, but these differences vanished at follow‐ups. Only PED‐t provided improvements in BDI, still with no between‐group difference. Totally, 30–50% of participants responded favorable to treatments, with no statistical between‐group difference.

**Discussion:**

Both treatments shared a focus on normalizing eating patterns, correcting basic self‐regulatory processes and reducing idealized aesthetic evaluations of self‐worth. The results point to the PED‐t as an alternative to CBT for BN and BED, although results are limited due to compliance and dropout rates. Replications are needed by independent research groups as well as in more clinical settings.

## 1 INTRODUCTION

Sustained recovery from bulimia nervosa (BN) or binge‐eating disorder (BED) requires that a treatment focuses on the overvaluation of controlling body weight and food intake, and the affective dysregulation represented by binge eating with or without purging (Fairburn et al., 1995). Cognitive behavior therapy (CBT) is empirically supported as effective in alleviating behavioral and cognitive maintaining factors (Hay, 2013; Linardon, Fairburn, Fitzsimmons‐Craft, Wilfley, & Brennan, 2017), and is currently held as the preferred therapy for BN and BED (Hay, 2013; Linardon et al., 2017; Linardon & Wade, 2018). Still, more than 60% do not fully abstain from symptoms after CBT (Linardon & Wade, 2018). High rates of premature treatment termination (Linardon, Hindle, & Brennan, 2018) and relapses (Södersten, Bergh, Leon, Brodin, & Zandian, 2017) may be due to insufficient delivery (Mulkens, de Vos, de Graaff, & Waller, 2018; Waller, Stringer, & Meyer, 2012), and patient's clinical heterogeneity beyond what is captured by the diagnostic criteria. Hence, the efficacy of new treatment options needs to be explored to reach out to persons with BN or BED who, for some reason, do not benefit from CBT or other kind of psychological treatments.

Physical activity is found to improve cognitive function, self‐regulation and positive affect (Buckley, Cohen, Kramer, McAuley, & Mullen, 2014; Lambourne & Tomporowski, 2010; Ludwig & Rauch, 2018; Mullen & Hall, 2015; Oaten & Cheng, 2006) as well as self‐esteem, self‐perception, self‐efficacy, global self‐worth and overall quality of life (Dupuy et al., 2015; Fox, 1999; Haugen, Säfvenbom, & Ommundsen, 2011; Oaten & Cheng, 2006; Vancampfort et al., 2014). In addition, regular physical activity is found to alleviate symptoms of anxiety and depression (Fox, 1999; Rosenbaum, Tiedemann, Sherrington, Curtis, & Ward, 2014; Stubbs et al., 2018). Such effects may be explained both neurobiological, psychosocial, and behavioral changes (Ashdown‐Franks et al., 2019; Lubans et al., 2016; Mullen & Hall, 2015). If physical activity is included in the treatment of BN and BED, such changes may be relevant to expect as both disorders are acknowledged as cognitive and neurobiological in nature (Donnelly et al., 2018; Grant & Chamberlain, 2019). Both immediate and longitudinal effects from moderate intensity physical activity have shown positive cognitive effect by improving eating behavior and self‐regulation in healthy students (Lowe, Hall, Vincent, & Luu, 2014; Oaten & Cheng, 2006). Noncompensatory physical activity is also associated with less psychopathology in persons with BED or BN (Kerrigan, Lydecker, & Grilo, 2019). However, physical activity is rarely incorporated in the treatment of BN (Bratland‐Sanda et al., 2009; Quesnel et al., 2018). One possible reason may be the fact that dysfunctional exercise affects approximately 20–40% of persons with BN (Dalle Grave, Calugi, & Marchesini, 2008), a behavior explained by the need for affect regulation, or in an urge to control body weight and shape (Meyer & Taranis, 2011). Clinicians may deem it counterintuitive to increase behaviors that are understood as BN‐symptoms, hence the reluctance in incorporating physical activity in treatment of eating disorders. However, such opinions and practices lack empirical evidence. On the contrary, in a previous randomized controlled trial (RCT) from our research group, we found that *therapist guided* group‐based physical activity was equal to group‐CBT in reducing binge eating, purging and pursuit of thinness in women with BN at the end of treatment, and even superior to CBT at 18 months follow‐up (Sundgot‐Borgen, Rosenvinge, Bahr, & Schneider, 2002). Moreover, uncontrolled studies indicate that physical activity reduces binge eating and body weight (Vancampfort et al., 2013), and increases self‐efficacy in treatment of BED (Vancampfort et al., 2014). Additionally, a recent systematic review identified beneficial effects from physical activity on treatment of BED (Blanchet et al., 2018). The weak empirical support for dietary counseling as a single treatment option for BN (Laessle et al., 1991; Sundgot‐Borgen et al., 2002) and BED (Hay, 2013) does not exclude the possibility that such counseling may amplify other treatment approaches such as CBT (Hsu et al., 2001). We argue that to combine dietary therapy with guided physical activity (physical exercise and dietary therapy, PED‐t) may represent an effective, alternative treatment option for persons with BN and BED. This combination has to our knowledge, never been tested against a well‐established psychological treatment using a RCT design.

We thus hypothesized that PED‐t is comparable to CBT in alleviating BN and BED‐ as well as depressive symptoms, and in increasing subjective well‐being and improving psychosocial impairment (Mathisen et al., 2017). We also hypothesized that the PED‐t would result in less drop out, and produce a more rapid treatment response than CBT.

## 2 METHODS

### 2.1 Study design, sample, and recruitment procedures

This RCT recruited women with BN or BED for outpatient treatment over 16 weeks arranged at the Norwegian School of Sport Sciences, in Oslo (Norway) during 2014–2017. All outcomes were measured by self‐reports at baseline (T1), after treatment (T2), and at 6‐ (T3), 12‐ (T4) and 24‐months (T5) posttreatment. Additionally, an experienced clinician assessed by interview the diagnostic criteria for BN and BED according to the definition by DSM‐5 (American Psychiatric Association, 2013); that is, an overvaluation of controlling body weight, −shape and food intake, and with frequency of symptomatology (binge eating and/or purging) at least once per week.

Women 18–40 years of age and with a DSM‐5 diagnosis of BN or BED (all categories of severity), and a BMI between 17.5 and 35 kg m^−2^ were eligible. The gender restriction and age range reflected the intention to increase treatment group homogeneity, while the upper BMI value was chosen because the manual‐based exercise program did not allow individual adjustments for reasons related to severe obesity. Considering the need to comply with a weekly, repetitive exercise program with high impact loading (i.e., interval running and weightlifting exercises like squats and walking lunges), being the manual, which PED‐t relies on, we did not find it safe to include persons with a BMI above 35. A comorbid axis I psychiatric disorder that precluded the eating disorder focused treatment and needed more extensive care was excluded. More thorough description of inclusion and exclusion criteria is provided in the open‐access published protocol (Mathisen et al., 2017).

Participants were recruited through general practitioners (GP's), magazines, newspapers, national TV, social media, posters, handouts, and through webpages of the national patient organizations for EDs. We interviewed participants by phone 2–6 weeks prior to the baseline measures and randomization, and all participants and their GP's had to sign an informed consent before final inclusion.

### 2.2 Randomization and masking

Two researchers not affiliated with the study produced the randomization list (by http://www.randomizer.org) with block size of eight, and concealed allocation to either group CBT or group PED‐t, and they performed allocation by listing the randomization results by id‐number. The project leader conveyed the allocation to the participants after they had completed the baseline measures. Participants recruited during ongoing treatment or who for some reason were unable to enter therapy immediately were put on a waitlist for 16 weeks and thereafter randomized to one of the two groups. This group served as a reference group for the two therapies at pre‐ and post‐treatment. All outcomes, anonymously coded by ID‐number, were transferred to SPSS files by blinded statistical staff before the final statistical analyses.

### 2.3 Procedure

The 16‐weeks treatments consisted of 20 group sessions, each of 90–120 min duration with 5–8 participants in each group.

The CBT manual rests on a transdiagnostic model positing generic core ED‐characteristics across ED‐diagnoses (Fairburn et al., 2009). The CBT‐therapy was based on Fairburn's individual CBT‐E therapy, but adapted for groups as described previously (Fairburn, 2008). This means that the treatment followed the CBT‐E protocol and it was designed to mirror the 20‐week treatment. The group meetings followed a similar format to that used in individual treatment and covered the exact same material. In advance of each meeting the patient met the therapist to be weighed and for a brief review of their monitoring records. The therapy consisted of 1–2 weekly therapy sessions of 90 minutes each, and the manual outlined four stages: (a) engagement and behavioral change, (b) monitoring and evaluating progress, (c) addressing the core ED‐ pathology, and (c) relapse prevention. Homework comprised registration of meals, thoughts and emotions during meal situations, and behavioral experiments. Clinical psychologists certified in CBT, with at least 10 years of experience, were conducting this therapy. The therapists were instructed and led by one senior therapist (Ph.D., clinical psychologist and scientist) having more than 20 years of experience in CBT and in treatment of eating disorders.

The PED‐t manual combines progressive resistance training and high intensity interval running, with dietary therapy; that is, education, group discussions and exploration of behavioral and practical skills (Mathisen et al., 2017). The PED‐t follows the same structure as the CBT‐E; hence, 20 therapy sessions in which 60 min dietary therapy directly followed 45 min with supervised resistance exercise. The dietary part of the PED‐t was arranged as focused modules, that is: (a) meal frequency, portion sizes and the eating context, (b) detailed knowledge about energy and nutrient needs, and exercise physiology theory, and (c) presentation of a personal plan for the future. Homework in the PED‐t therapy consisted of two unsupervised exercise sessions per week (one resistance exercise and one interval running session, both following a prescribed, progressive program), and dietary tasks related to weekly topics (Mathisen et al., 2017). Physical exercise therapists and dietitians (M.Sc.) were responsible for this therapy, and they received supervision from an experienced therapist (Ph.D., dietitian, and exercise therapist).

A detailed account of the procedures, safety agenda and the treatment contents has been described previously (Mathisen et al., 2017). Previously described are also results with respect to effects on physical health and dysfunctional exercise routines (Mathisen et al., 2018; Mathisen, Sundgot‐Borgen, Rosenvinge, & Bratland‐Sanda, 2018), as well as feasibility and acceptability (Bakland et al., 2018; Bakland et al., 2019; Pettersen et al., 2017).

### 2.4 Outcomes

#### 2.4.1 Eating disorder examination questionnaire (EDE‐Q)—6.0

The EDE‐Q 6.0 (Cronbach's *α* = .87, T1 current study) validly assesses the frequency and severity of ED features to produce ED diagnoses according to the DSM‐5 (Fairburn, 2008). Apart from a global score, four subscales measure eating restraint (ER), eating concern (EC), shape concern (SC), and weight concern (WC). Mean (*SD*) global score for a Norwegian cohort of healthy female controls is 1.25 (1.10), while corresponding national clinical cutoff for probable BN and BED are 2.62, and 2.63, respectively (Rø, Reas, & Stedal, 2015). Reported binge‐eating episodes are based on scores from EDE‐Q item 14 (*reported binge eating with loss of control*). Reported purging episodes are based on scores from EDE‐Q item 16–18 (i.e.*, purging by self‐induced vomiting, use of laxatives, and driven/compulsive exercise*).

#### 2.4.2 Remission

Calculation of numbers in full remission is based on the EDE‐Q global score of the national cohort sample's normative value +1 *SD* (i.e., ≤2.35) (Rø et al., 2015), with concurrently abstinence from binge eating and weight compensatory behavior (i.e., 0 episodes last 28 days, as reported in EDE‐Q item 13–18). Calculation of numbers in partial remission is based on the EDE‐Q global score below the diagnostic specific cutoff (i.e., 2.62 in BN and 2.63 in BED) (Rø et al., 2015), with concurrently less binge eating and purging episodes defined in diagnostic criteria (i.e., <4 times last 28 days). Additionally, remission from BN or BED diagnoses was validated with an experienced ED‐therapist applying the DSM‐5 criteria in a clinical interview.

#### 2.4.3 Clinical impairment assessment (CIA 3.0)

The CIA 3.0 (Cronbach's *α* = .90, T1 current study) measures ED‐elicited personal, social, and cognitive impairment (Fairburn, 2008). The CIA consists of 16 items scored on a 4‐point Likert scale ranging from 0 (not at all) to 3 (a lot) during the past 28 days, with total scores ranging 0–48 (Bohn et al., 2008).

#### 2.4.4 Beck depression inventory (BDI‐Ia)

The BDI‐Ia (Cronbach's *α* = .86, based on T1) measures current (past 2 weeks) self‐reported symptoms of depression (Beck, Ward, Mendelson, Mock, & Erbaugh, 1961). It consists of 21 items scored with a 4‐point Likert scale ranging from 0 (not at all) to 3 (extreme). Total score range is 0–62, and scores above ≥21 is a recommended cutoff indicating a clinically significant episode of major depression (Beck et al., 1961).

#### 2.4.5 Satisfaction with life scale (SWLS)

The SWLS (Cronbach's *α* = .89, based on T1) is a short 5‐item scale measuring overall contentment with life compared to personal standards and expectations, using a 5‐point Likert scale ranging from 1 (never true) to 7 (always true) (Diener, 1994).

### 2.5 Dropout and lost to follow‐up

We defined dropouts as those who did not complete the treatment, or those who by other reasons did not provide posttreatment data. To reduce loss of therapy sessions and avoid early dropout, we contacted all participants who were missing from therapy sessions. Lost to follow‐up are those not attending follow‐up testing (T3–T5), typically for reasons like work/studies, moving too far away, injuries or illness, pregnancy, not willing to attend physically to the testing facilities, and not responding to email, mail or electronic questionnaires.

### 2.6 Statistical analyses

The power calculation for this trial is provided elsewhere (Mathisen et al., 2017). The SPSS version 24 was used for all analyses. Differences between diagnostic groups at baseline were explored with independent *t*‐tests or Mann–Whitney test. Attrition rates were analyzed separately for each time (posttreatment and follow‐ups, T2–T5) with independent *t*‐test or chi‐square analyzes as appropriate. A significance level of *p* < .05 was used for all bivariate comparisons.

All inferential tests defined the two therapy arms, CBT and PED‐t, as a between‐group factor, whereas the repeated posttest scores represented a within‐group factor. The low (*n* = 23) number of waitlist participants was excluded from group comparisons due to low statistical power, but are presented as a reference group in the figure and tables. Linear mixed regression models were built to estimate the coefficients for the between (PED‐t vs. CBT) and the within factors (baseline vs. any of the three posttest measures). This analysis yields relatively unbiased estimates despite drop out given that data are missing completely at random or missing at random. Moreover, it can be safely used without conducting beforehand multiple imputations (Twisk, de Boer, de Vente, & Heymans, 2013). Standard errors were estimated with the restricted maximum likelihood function. Type III *F*‐tests were used. The dependency in the four repeated outcome measures tended to decline across time, and hence an autoregressive model was fitted to the residual covariance matrix. The fixed factors were: *Group* (0‐PED‐t, 1‐CBT) representing the overall therapy difference, *Time* (repeated measures) representing change across measurements, and the *Group* × *Time* interaction in order to detect treatment differences at certain time points only. The between‐group analyses used the baseline values as a covariate to increase the statistical power (Egbewale, Lewis, & Sim, 2014). Differences between the therapy arms were examined with planned comparisons at each time point (least square difference tests). In addition, linear time trends were estimated by specifying time as a covariate instead of a fixed (and dichotomous) effect. The within‐group analyses included all four measurements in the *Time* factor. Due to the number of tests, differences with *p*‐values <.01 were considered as statistically significant.

A comparable statistical approach was used for the dichotomous outcome variables, replacing the analysis with a generalized linear model using a binominal distribution, a logit link function (reference coded 0), and the Satterthwaite approximation to settle a more appropriate degrees of freedom. For that reason, we provide 99% confidence bands for the estimated means.

Standardized Hedge's *g* effect‐sizes for continuous data were calculated as a ratio of the estimated mean difference (based on the mixed model) to the observed pooled *SD*s. Values around .2, .5 and .8 were interpreted as weak, medium and strong, respectively (Hedges & Olkin, 1985). Following the intention to analyze results according to a noninferiority trial, only effect‐sizes of at least 0.45 in differences between groups were considered clinical significant (Mathisen et al., 2017).

## 3 RESULTS

**Figure 1:**
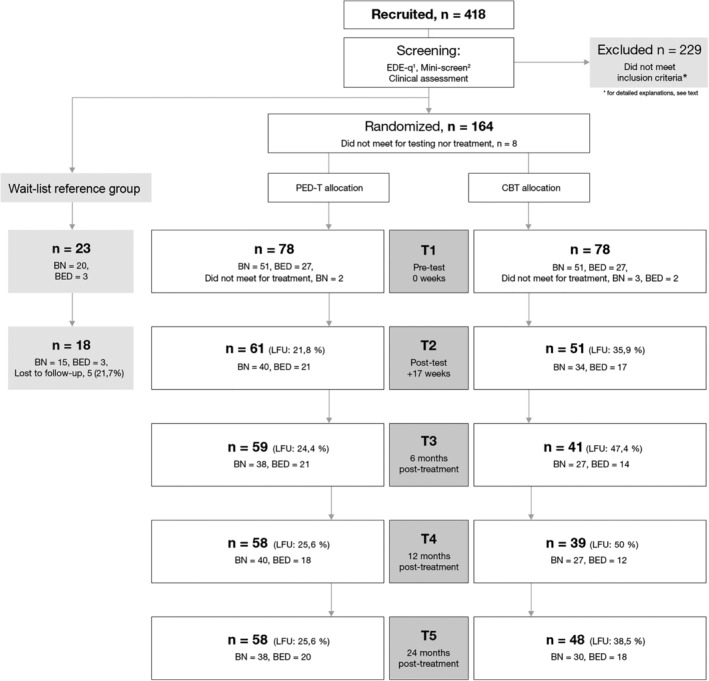
Recruitment, retention, and attrition during test periods. Overview of recruitment, screening, randomization and attendance to the baseline (T1), posttreatment (T2), and follow‐up measures (T3–T5). *Note:* PED‐t, physical exercise and dietary therapy; CBT, cognitive behavior therapy; BN, bulimia nervosa; BED, binge‐eating disorder; EDE‐Q, eating disorder examination questionnaire; LFU, lost to follow‐up; ^1^Fairburn and Beglin (2008); ^2^Sheehan et al. (1998)

Of the 418 women who responded during the recruitment period (March 2014–August 2016), 229 did not fulfill the inclusion criteria, 164 were randomized to therapy groups, and finally 76 and 73 initiated therapy in PED‐t and CBT, respectively (Figure 1). Reasons for being excluded were either general reasons (i.e., sex, age, BMI, physical injuries, living distance to treatment facility, *n* = 54), diagnostic reasons (i.e., no ED‐diagnosis or having AN, *n* = 54), having severe comorbidity (i.e., suicidality, posttraumatic stress disorder, obsessive–compulsive disorder, bipolarity, personality disorder, referred to inpatient treatment, *n* = 37), other self‐chosen reasons (i.e., did not find the time, did not want to attend anyway, *n* = 60), or other competing treatment (i.e., recently received CBT or preferred other concurrently offered therapy, *n* = 24).

The mean percentage (*SD*) attendance rate to therapy was 80.6% (11.4) in PED‐t, and 82.1% (45.7) in CBT. In PED‐t the compliance rate to exercise sessions, as supervised in therapy + unsupervised at home (being evaluated from attendance to therapy and evaluated from training diaries) was 69.8% and 56.7% for resistance‐ and interval exercises, respectively. There were significant differences between diagnoses at baseline, with higher body weight and BMI among those with BED, and higher EDE‐Q global score and number of purging episodes among those with BN (Table 1).

**Table 1:** Demographics of participants

	Bulimia nervosa (*n* = 117)	Binge‐eating disorder (*n* = 55)	PED‐t (*n* = 76)	CBT (*n* = 73)	Reference (*n* = 23)
Age, years	27.2 (5.6)	29.0 (6.1)	28.2 (6.2)	27.7 (5.3)	26.5 (5.6)
BW, kg	66.0 (12.2)	81.9 (15.1)*	71.7 (16.0)	71.4 (14.4)	67.2 (14.1)
BMI, kg/m^2^	23.5 (3.8)	28.9 (5.0)*	25.3 (5.1)	25.5 (4.7)	24.1 (4.9)
Illness duration, years	12.0 (6.6)	13.0 (8.2)	13.0 (7.5)	12.1 (6.7)	10.6 (7.4)
EDE‐Q global score	3.79 (0.9)	3.49 (0.9)**	3.70 (0.8)	3.67 (1.0)	3.81 (1.0)
Binge‐eating episodes^a^	10.0 10.3, 14.1	10.0 8.5, 14.8	10.0 9.7, 15.0	9.0 9.0, 13.6	10.0 8.6, 17.7
Self‐induced vomiting^a^	5.0 9.0, 20.1	0.0* 0.0, 1.7	2.0 5.0, 20.6	0.0 4.8, 12.8	0.0 0.1, 10.9
Laxative use^a^	0.0 0.5, 1.8	0.0*** −0.4, 1.7	0.0 −0.01, 1.6	0.0 0.45, 2.4	0.0 −0.01, 7.1
Driven exercise^a^	10.0 9.3, 13.9	4.5**** 5.0, 9.6	7.5 8.2, 13.4	6.0 6.4, 10.1	10.0 6.1, 23.8

*Note:* Baseline demographic and clinical information presented per diagnosis, and per therapy‐ or reference group. Results are mean (*SD*), except binge eating, and purging episodes which are median (Ci_95_).

Abbreviations: BED, binge‐eating disorder; BMI, body mass index; BN, bulimia nervosa; BW, body weight; CBT, cognitive behavior therapy; EDE‐Q global, eating disorder examination questionnaire; PED‐t, physical exercise and dietary therapy.

a Episodes last 28 days.

*p < .001; **p = .04; ***p = .03; ****p = .014.

The numbers of dropout from treatment were not significantly different across groups or ED diagnoses. Among those who dropped out at posttest (*n* = 37) a higher mean (CI_95_) baseline score for depression was found compared to completers (*n* = 112), (3.36 _6_._19, 1_._43_, *g* = .44, *p* = .02). Significantly more CBT‐participants were lost to follow‐up (LFU) at 6‐months follow‐up (PED‐t = 19 (24.4%), CBT = 37 (47.4%), *p* = .005), 12‐months follow‐up (PED‐t = 20 (25.6%), CBT = 39 (50.0%), *p* = .003), and 24‐months follow‐up (PED‐t = 20 (25.6%), CBT = 25 (38.5%), *p* = .03). Overall, there was no difference in LFU across the BN and BED diagnoses (*p* > .02); however, more with BN in CBT were LFU at all follow‐ups compared to BN in PED‐t (*p* < .03). There were no differences (*p* > .06) between attending participants at 6‐months follow‐up (*n* = 100, 64.1%) and LFUs (*n* = 56, 35.9%) with respect to the scoring at posttest, as was the case at 12‐months follow‐up with respect to 6‐months follow‐up scores (attendees; *n* = 97, 62.2%; LFU; *n* = 59, 37.8%) (*p* > .05), and at 24‐months follow‐up with respect to 12‐months follow‐up scores (attendees; *n* = 106, 61.6%; LFU: *n* = 43, 25%) (*p* > .05).

### 3.1 Effects of PED‐t and CBT on ED symptoms (EDE‐Q)

#### 3.1.1 Change in EDE‐Q symptom scores from baseline to post and follow‐up tests

Compared to baseline, both therapy groups improved in EDE‐Q global score at posttreatment and at all follow‐ups (PED‐t: T2, *g* = 1.36, *p* < .001; T3, *g* = .86, *p* < .001; T4, *g* = 1.17, *p* < .001; T5, *g* = 1.21, *p* < .001, and CBT: T2, *g* = .93, *p* < .001; T3, *g* = .63, *p* < .001; T4, *g* = 1.21, *p* < .001; T5, *g* = .99, *p* < .001). The same relate to EDE‐Q subscales weight concern (PED‐t: T2, *g* = 1.02, *p* < .001; T3, *g* = .61, *p* < .001; T4, *g* = .91, *p* < .001; T5, *g* = .87, *p* < .001, and CBT: T2, *g* = .60, *p* < .001; T3, *g* = .41, *p* = .004; T4, *g* = .82, *p* < .001; T5, *g* = .64, *p* < .001), shape concern (PED‐t: T2, *g* = 1.04, *p* < .001; T3, *g* = .70, *p* < .001; T4, *g* = .99, *p* < .001; T5, *g* = 1.03, *p* < .001, and CBT: T2, *g* = .50, *p* < .001; T3, *g* = .74, *p* < .001; T4, *g* = .82, *p* < .001; T5, *g* = .84, *p* < .001), eating restraint (PED‐t: T2, *g* = 1.24, *p* < .001; T3, *g* = .80, *p* < .001; T4, *g* = .92, *p* < .001; T5, *g* = .90, *p* < .001, and CBT: T2, *g* = .87, *p* < .001; T3, *g* = .72, *p* < .001; T4, *g* = 1.24, *p* < .001; T5, *g* = .93, *p* < .001)., and eating concern (PED‐t: T2, *g* = 1.28, *p* < .001; T3, *g* = .97, *p* < .001; T4, *g* = 1.28, *p* < .001; T5, *g* = 1.51, *p* < .001, and CBT: T2, *g* = .91, *p* < .001; T3, *g* = .56, *p* < .001; T4, *g* = 1.25, *p* < .001; T5, *g* = 1.32, *p* < .001). Additionally, both therapy groups reported a significant alleviation of binge eating from baseline to posttreatment (*p* < .001 in both groups), and from baseline to each follow‐up (PED‐t T3 *p* < .001; T4 *p* < .001; T5 *p* = .003, and CBT T3 *p* < .001; T4 *p* = .007; T5 *p* < .001). Both therapy groups also reported reduction in episodes of self‐induced vomiting from baseline to posttreatment (*p* = .003 in PED‐t and *p* = .002 in CBT) and from baseline to most follow‐ups' (PED‐t T3 *p* = .004; T5 *p* < .001, and CBT T4 *p* = .005; T5 *p* < .001), except at T4 in PED‐t (*p* = .02) and at T3 in CBT (*p* = .12). There were no significant changes in the number of episodes with laxative use (*p* > .02), but both groups reduced the number of episodes with driven exercise from baseline to posttreatment (*p* < .001 in both groups), and from baseline to all follow‐ups (PED‐t T3 *p* < .001; T4 *p* = .005; T5 *p* < .001, and CBT T4 *p* < .001; T5 *p* = .007), except at T3 in CBT (*p* = .09).

#### 3.1.2 Therapy effect differences in EDE‐Q symptom scores (between analyses with baseline as covariate)

The fixed effect of *Time* was significant (*F*_3,282_ = 8.04, *p* < .001) indicating a general improvement across the follow‐ups. However, the more important *Group* and Group * Time effects were not significant (*F*_1,134_ = 2.79, *p* = .097; *F*_3,282_ = 2.47, *p* = .062, respectively). Planned comparison tests nevertheless indicated an advantage of PED‐t above CBT at the first posttest (T2) exclusively in EDE‐Q global score (*g* = 0.54, *p* = .004) and the EDE‐Q subscale “body weight concern” (*g* = 0.53, *p* = .003). Analyzing *Time* as a linear trend indicated a general decline across the follow‐ups (*b*_time_ = −.177, *p* = .025), but it was not significantly different by *Group* (*b*_time*group_ = .146, *p* = .115).

Neither the fixed effect of *Time*, *Group*, nor *Group* * *Time* effects were significant for binge eating and purging episodes, and no planned between‐group comparison test were significant either.

### 3.2 Effects of PED‐t and CBT on full‐ and partial remission

#### 3.2.1 Full remission

**Figure 2:**
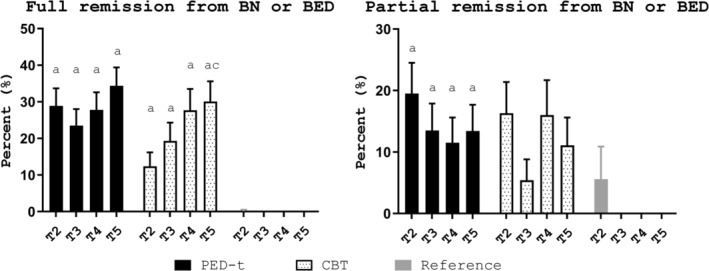
Remission from diagnosis. Estimated mean percent (SE) in each group in full remission (left) and partial remission (right) at posttreatment (T2) and follow‐ups (T3‐T5). *Note:* PED‐t, physical exercise and dietary therapy; CBT, cognitive behavior therapy; T2, posttreatment; T3, 6‐month posttreatment; T4, 12‐month posttreatment; T5, 24 months follow‐up; a, significant change from baseline, *p* < .01; c, significant change from T2, *p* < .003

In both therapy groups, the number of participants in remission was significantly higher posttreatment (PED‐t *p* < .001; CBT *p* = .001) and at follow‐ups (T3‐T5: PED‐t *p* < .001; CBT *p* < .001) compared to baseline, with no differences between the groups (T2 *p* = .03; T3 *p* = .6; T4 *p* = 1.0; T5 *p* = .7) (Figure 2).

#### 3.2.2 Partial remission

The number in partial remission in the PED‐t group was higher posttreatment (*p* < .001) and all follow‐ups (T3 *p* = .006; T4 *p* = .01; T5 *p* = .005), compared to baseline. The changes in the CBT group approached, but were not significantly different posttreatment (*p* = .02), with no differences to baseline at follow‐ups (T3 *p* = .7; T4 *p* = .04; T5 *p* = .2). There was no difference in numbers in partial remission between the two groups at any time (T2 *p* = .5; T3 *p* = .2; T4 *p* = .6; T5 *p* = .7) (Figure 2).

### 3.3 Effects of PED‐t and CBT on clinical impairment (CIA) from ED

#### 3.3.1 Change in CIA scores from baseline to post and follow‐up tests

Compared to baseline, both therapy groups had reduced CIA score posttreatment (*p* < .001) and at follow‐up tests (*p* < .001, Table 2). The effect sizes were strong for PED‐t, and ranged between medium and strong for CBT.

**Table 2:** Satisfaction with Life Scale (SWLS), Beck Depression Inventory (BDI), and Clinical Impairment Assessment (CIA)

								Between effects, *p*‐value, effect size (*g*)			
		T1_CI .99_		T1–T2	T1–T3	T1–T4	T1–T5	T2	T3	T4	T5
SWLS	PED‐t	16.50 14.37–18.63	Δ (CI_99_)	4.60 2.73, 6.46 *p* < .001 *g* = 0.69	2.92 1.00, 4.84 *p* < .001 *g* = 0.44	3.81 1.92, 5.69 *p* < .001 *g* = 0.64	5.04 3.16, 6.91 *p* < .001 *g* = 0.76				
	CBT	16.63 14.46–18.80	Δ (CI_99_)	2.27 0.26, 4.28 *p* = .004 *g* = 0.53	n.s.	2.53 0.32, 4.74 *p* = .003 *g* = 0.60	3.98 1.90, 6.10 *p* < .001 *g* = 0.63	n.s	n.s	n.s	n.s.
	Reference	16.17 12.31–20.04	Δ (CI_99_)	n.s.	–	–	–	–	–	–	–
BDI	PED‐t	16.50 13.67–19.28	Δ (CI_99_)	−6.73 −9.93, −3.54 *p* < .001 *g* = 0.93	−4.95 −8.20, −1.71 *p* < .001 *g* = 0.66	n.s.	−4.5 −7.72, −1.34 *p* < .001 *g* = 0.47				
	CBT	14.56 11.70–17.42	Δ (CI_99_)	n.s.	n.s.	n.s.	n.s.	n.s.	n.s.	n.s	n.s.
	Reference	18.52 13.45–23.59	Δ (CI_99_)	n.s.	–	–	–	–	–	–	–
CIA	PED‐t	28.22 24.87–31.57	Δ (CI_99_)	−13.97 −17.68, −10.26 *p* < .001 *g* = 1.18	−12.63 −16.41, −8.86 *p* < .001 *g* = 1.07	−12.46 −16.30, −8.61 *p* < .001 *g* = 1.01	−16.06 −19.85, −12.28 *p* < .001 *g* = 1.36				
	CBT	26.90 23.52–30.30	Δ (CI_99_)	−7.05 −10.99, −3.11 *p* < .001 *g* = 0.78	−5.99 −10.20, −1.78 *p* < .001 *g* = 0.56	−11.71 −16.17, −7.30 *p* < .001 *g* = 1.01	−10.88 −15.25, −6.51 *p* < .001 *g* = 1.08	−6.20 −11.88, −0.52 *p* = .005 *g* = 0.50	n.s.	n.s	n.s.
	Reference	29.99 23.91–36.10	Δ (CI_99_)	n.s.	–	–	–	–	–	–	–

*Note:* Estimated mean scores (Ci_99_) according to group affiliation (PED‐t, CBT, or reference) and time (T1–T5).

Abbreviations: between, difference between groups (adjusted for baseline) at any of the four posttests (T2–T5); CBT, cognitive behavior therapy; CI_99_, 99% confidence interval; EDE‐Q, eating disorder examination questionnaire; *g*, effect size of Hedges *g*; n.s., nonsignificant; PED‐t, physical exercise and dietary therapy; REF, reference group; T1, baseline measure; T2, posttreatment; T3, 6‐month follow‐up posttreatment; T4, 12‐month follow‐up posttreatment; T5, 24‐month follow‐up posttreatment; within, change from baseline to any of the four posttests (T2–T5); Δ, change.

#### 3.3.2 Therapy effect differences in CIA scores (between analyses with baseline as covariate)

The fixed effect *Group* (*F*_1,118_ = 6.86, *p* = .010) was significant. The *Time* (*F*_2,230_ = 3.20, *p* < .024) and *Group* * *Time* (*F*_3,230_ = 1.83, *p* = .143) effects were not significant. The planned comparison tests (between‐effects column in Table 2) showed an advantage of PED‐t above CBT posttreatment and at first follow‐up (*g* = 0.55, *p* = .005) (T2 and T3 in Table 2), but not at later follow‐ups (T4‐T5). Analyzing time as a linear trend indicated a decline across follow‐up, still not significant (*b*_time_ = −1.36, *p* = .068), and neither did it significantly co‐vary by group (*b*_time*group_ = .63, *p* = .524).

### 3.4 Effects of PED‐t and CBT on depressive symptoms (BDI)

#### 3.4.1 Change in BDI scores from baseline to post and follow‐up tests

Compared to baseline, participants in PED‐t displayed reduced BDI scores posttreatment (p < .001) and at follow‐ups (T3 *p* < .003 and T5 *p* < .001, Table 2). The effect sizes ranged between medium and strong. There were no significant changes in BDI scores among participants in CBT. The estimated mean number of participants (_Ci 99%_) with BDI score above clinical cutoff was reduced in PED‐t at T2 with a marginal statistical significance (from 27.8% _16.6, 42.6_ to 13.9% _5.9, 29.3_, *p* = .014), but with no further differences to baseline at follow‐ups. There were no significant changes in number of participants with scores above cutoff in CBT (T1 = 24.8% _14.1, 39.8_).

#### 3.4.2 Therapy effect differences in BDI scores (between analyses with baseline as covariate)

The fixed effect *Group* was significant (*F*_1,124_ = 7.21, *p* = .008), whereas *Time* (*F*_3,256_ = 2.99, *p* < .031) and *Group* * *Time* (*F*_3,256_ = .71, *p* = .546) was not. The planned comparison tests showed a tentative advantage from PED‐t above CBT posttreatment (*p* = .016), but not in the remaining posttests. The linear trend analysis of Time was not significant (*b*_time_ = −.831, *p* = .164), and did not significantly co‐vary with group (*b*_time*group_ = .159, *p* = .842). We found no differences between groups by time in percentage of participants scoring above BDI clinical cutoff (*p* > .11).

### 3.5 Effects of PED‐t and CBT on subjective well‐being (SWLS)

#### 3.5.1 Change in SWLS scores from baseline to post and follow‐up tests

Compared to baseline, both therapy groups' improved SWLS scores posttreatment (*p* < .003) and at follow‐ups (T3‐T5 *p* < .01, Table 2). The effect sizes were medium in both groups.

#### 3.5.2 Therapy effect differences in SWLS scores (between analyses with baseline as covariate)

The fixed effect of *Group* (*F*_1,137_ = 2.50, *p* = .117) and of *Group* * *Time* (*F*_3,276_ = 1.03, *p* = .380) was not significant, whereas the effect of *Time* approached significance (*F*_3,276_ = 1.03, *p* < .022). The trend analysis of Time was not significant (*b*_time_ = .522, *p* = .138; *b*_time*group_ = −.383, *p* = .418).

## 4 DISCUSSION

Overall, we found support to our hypothesis, as the PED‐t performed equal to CBT in alleviating symptoms of BN, BED, as well as in improving subjective well‐being and psychosocial impairment. In addition, PED‐t reduced symptoms of depression. We also found support for the hypothesis that the PED‐t produced a more rapid treatment response (i.e., the T1 to T2 change in EDE‐Q global score, EDE‐Q subscale “body weight concern”, CIA and a tentative difference in BDI), however, contradicting our hypothesis, was the lack of difference between groups in drop out from treatment.

The effect size of changes within groups was with medium to strong effects, hence, implying changes with important clinical impact. This also relates to the acute differences identified between groups posttreatment in EDE‐Q global score, body weight concern and clinical impairment. Nevertheless, both therapies performed well in an outpatient group setting, with a summarized response rate (i.e., the total of participants that either reached full or partial remission) to therapy of ~50% after PED‐t and ~30% after CBT. This corresponds well with previous findings on treatment outcome from group‐CBT, that is, ~0–52% remission after group‐CBT (Linardon, 2018; Linardon, Messer, & Fuller‐Tyszkiewicz, 2018; Linardon & Wade, 2018).

Early symptom changes promote treatment success and predict a favorable long‐term outcome (Graves et al., 2017). An important asset of CBT compared to other treatments of EDs, is that CBT produces such early changes (Agras, Walsh, Fairburn, Wilson, & Kraemer, 2000; Fairburn, Jones, Peveler, Hope, & O'Connor, 1993; Hay, 2013; Södersten et al., 2017; Wilson, Fairburn, Agras, Walsh, & Kraemer, 2002), thus leading to a shorter time of suffering from EDs. A similar asset may be transferred to the PED‐t compared to CBT in terms of less impairment, and a remission from the BN and BED diagnoses. It is not clear why the previously described rapid effect from CBT was not found in our study, but one explanation could be attributed to the randomization procedure. Considering the study setting and how the study was profiled as a physical activity trial, the rapid effect may reflect the motivation, interest and preference for the new therapy (PED‐t) (and not the CBT) among those who chose to participate. If so, the rapid treatment response may not be expected to occur in a non‐RCT context.

Although alleviations of core ED symptoms is necessary for a treatment success, improvements in secondary outcomes is important to consolidate symptom improvement and to strengthen positive circuits created by a rapid symptom change. The participants' subjective well‐being was improved in both therapies, and about 60–70% of all participants rated their well‐being equal to or above average during follow‐up, which is close to the frequency of 83% in national cohort studies (Diener & Diener, 2009). Additionally, the positive effect from PED‐t on depression scores is in line with other studies examining effects of physical activity in persons diagnosed with depression (Fox, 1999; Rosenbaum et al., 2014), and summarized evidence holds physical activity at least as effective as psychological or medical interventions (Stubbs et al., 2018). Excluding severe depression may account for low BDI‐scores throughout, however findings of symptoms of depression in the current sample were comparable to previous reports in similar populations (Keski‐Rahkonen & Mustelin, 2016).

Both the CBT and the PED‐t shared a focus on enhancing self‐regulative mechanisms and in particular with respect to eating as well as changing negative body and shape self‐evaluations and how such evaluations used to be detrimental for self‐worth. It is rather intriguing that these endpoints were successfully approached through very dissimilar means, that is, by verbal behaviors (CBT) and practical experiences of body functionality (PED‐t). The dissimilarity makes future search for possible common mechanisms of change highly interesting. Neurobiological and cognitive factors may be considered, given that these factors have been associated with effects of physical activity (Lubans et al., 2016; Mullen & Hall, 2015) and that they constitute important core features of BN and BED (Donnelly et al., 2018).

Strengths to this study are the RCT‐design with 24 months follow‐up, the equal length and duration of the treatments, and the unlikely diffusion of effects from previous or concurrent treatments. Limitations in need to be mentioned are the low compliance rate to PED‐t exercise module, the large number of CBT‐participants lost to follow‐ups as well as the failure to control for therapist factors, and for other kind of treatments that may have been sought in the follow‐up period. Concerning the loss of CBT‐participants, we argue the statistical model handles this most favorable, with full information maximum likelihood methods reducing any risk of biased data (Witkiewitz et al., 2014). In addition, generalizability is limited to comparable gender, BMI and age, and may also have been hampered by the fact that severe comorbidity was excluded. Still, the level of symptom distress do suggest comparability to the level of anxiety‐, mood‐, and personality disorders among persons with BN and BED (Friborg et al., 2014; Godart et al., 2007; Keski‐Rahkonen & Mustelin, 2016; Martinussen et al., 2017; Swanson, Crow, Le Grange, Swendsen, & Merikangas, 2011). Finally, data on treatment fidelity are presently unavailable, and the recruitment information for this trial (i.e., offering therapy with physical activity, and a limitation of BMI range) may have resulted in a selected sample of patients.

### 4.1 Conclusion and implications

The effects of PED‐t are overall on par with the well‐established CBT for BN and BED. Its focus on physical exercise and diet may appeal to many sufferers. In addition, PED‐t may represent an alternative for those who do not want CBT, or in cases where CBT is unavailable. This can be accommodated given the high availability of professionals with expertise in exercise medicine and nutrition compared to CBT‐certified therapists. Following the logic behind the construction of the PED‐t it may be worthwhile to test the incremental effects of combining PED‐t and CBT. Replications of the present findings are needed both by independent research groups and within new and preferably more clinical contexts. Equally needed is the exploring of mechanisms of change.

## Data Availability

The data that support the findings of this study are available on request from the corresponding author. The data are not publicly available due to privacy or ethical restrictions.

## References

[eat23228-bib-0001] Agras, W. , Walsh, B. , Fairburn, C. G. , Wilson, G. , & Kraemer, H. C. (2000). A multicenter comparison of cognitive‐behavioral therapy and interpersonal psychotherapy for bulimia nervosa. Archives of General Psychiatry, 57(5), 459–466. 10.1001/archpsyc.57.5.459 10807486

[eat23228-bib-0002] American Psychiatric Association . (2013). Diagnostic and statistical manual of mental disorders (DSM‐5®) (5th ed.). Arlington: American Psychiatric Pub.

[eat23228-bib-0003] Ashdown‐Franks, G. , Firth, J. , Carney, R. , Carvalho, A. F. , Hallgren, M. , Koyanagi, A. , … Stubbs, B. (2019). Exercise as medicine for mental and substance use disorders: A meta‐review of the benefits for neuropsychiatric and cognitive outcomes. Sports Medicine, 50, 151–170. 10.1007/s40279-019-01187-6 31541410

[eat23228-bib-0004] Bakland, M. , Rosenvinge, J. H. , Wynn, R. , Sundgot‐Borgen, J. , Mathisen, T. F. , Liabo, K. , … Pettersen, G. (2019). Patients' views on a new treatment for bulimia nervosa and binge eating disorder combining physical exercise and dietary therapy (the PED‐t). A qualitative study. Eating Disorders, 27, 1–18. 10.1080/10640266.2018.1560847 30664397

[eat23228-bib-0005] Bakland, M. , Sundgot‐Borgen, J. , Wynn, R. , Rosenvinge, J. H. , Stornæs, A. V. , & Pettersen, G. (2018). Therapists' experiences with a new treatment combining physical exercise and dietary therapy (the PED‐t) for eating disorders: An interview study in a randomised controlled trial at the Norwegian School of Sport Sciences. BMJ Open, 8(1), e019386 10.1136/bmjopen-2017-019386 PMC5781022 29330176

[eat23228-bib-0006] Beck, A. T. , Ward, C. H. , Mendelson, M. M. , Mock, J. J. , & Erbaugh, J. J. (1961). An inventory for measuring depression. Archives of General Psychiatry, 4(6), 561–571. 10.1001/archpsyc.1961.01710120031004 13688369

[eat23228-bib-0007] Blanchet, C. , Mathieu, M.‐È. , St‐Laurent, A. , Fecteau, S. , St‐Amour, N. , & Drapeau, V. (2018). A systematic review of physical activity interventions in individuals with binge eating disorders. Current Obesity Reports, 7(1), 76–88. 10.1007/s13679-018-0295-x 29460067

[eat23228-bib-0008] Bohn, K. , Doll, H. A. , Cooper, Z. , O'Connor, M. , Palmer, R. L. , & Fairburn, C. G. (2008). The measurement of impairment due to eating disorder psychopathology. Behaviour Research and Therapy, 46(10), 1105–1110. 10.1016/j.brat.2008.06.012 18710699 PMC2764385

[eat23228-bib-0009] Bratland‐Sanda, S. , Rosenvinge, J. H. , Vrabel, K. A. R. , Norring, C. , Sundgot‐Borgen, J. , Rø, Ø. , & Martinsen, E. W. (2009). Physical activity in treatment units for eating disorders: Clinical practice and attitudes. Eating and Weight Disorders—Studies on Anorexia, Bulimia and Obesity, 14(2), e106–e112 10.1007/bf03327807 19934623

[eat23228-bib-0010] Buckley, J. , Cohen, J. D. , Kramer, A. F. , McAuley, E. , & Mullen, S. P. (2014). Cognitive control in the self‐regulation of physical activity and sedentary behavior. Frontiers in Human Neuroscience, 8(747), 1–15. 10.3389/fnhum.2014.00747 25324754 PMC4179677

[eat23228-bib-0011] Dalle Grave, R. , Calugi, S. , & Marchesini, G. (2008). Compulsive exercise to control shape or weight in eating disorders: Prevalence, associated features, and treatment outcome. Comprehensive Psychiatry, 49(4), 346–352. 10.1016/j.comppsych.2007.12.007 18555054

[eat23228-bib-0012] Diener, E. (1994). Assessing subjective well‐being: Progress and opportunities. Social Indicators Research, 31(2), 103–157. 10.1007/bf01207052

[eat23228-bib-0013] Diener, E. , & Diener, M. (2009). Cross‐cultural correlates of life satisfaction and self‐esteem In Diener E. (Ed.), Culture and well‐being: The collected works of Ed Diener (pp. 71–91). Dordrecht, Netherlands: Springer.

[eat23228-bib-0014] Donnelly, B. , Touyz, S. , Hay, P. , Burton, A. , Russell, J. , & Caterson, I. (2018). Neuroimaging in bulimia nervosa and binge eating disorder: A systematic review. Journal of Eating Disorders, 6, 3 10.1186/s40337-018-0187-1 29468065 PMC5819247

[eat23228-bib-0015] Dupuy, O. , Gauthier, C. J. , Fraser, S. A. , Desjardins‐Crèpeau, L. , Desjardins, M. , Mekary, S. , … Bherer, L. (2015). Higher levels of cardiovascular fitness are associated with better executive function and prefrontal oxygenation in younger and older women. Frontiers in Human Neuroscience, 9(66), 1–12. 10.3389/fnhum.2015.00066 25741267 PMC4332308

[eat23228-bib-0016] Egbewale, B. E. , Lewis, M. , & Sim, J. (2014). Bias, precision and statistical power of analysis of covariance in the analysis of randomized trials with baseline imbalance: A simulation study. BMC Medical Research Methodology, 14(1), 49 10.1186/1471-2288-14-49 24712304 PMC3986434

[eat23228-bib-0017] Fairburn, C. , & Beglin, S. (2008). Eating disorder examination questionnaire (EDE‐Q 6.0) In Fairburn C. (Ed.), Cognitive behavior therapy and eating disorders. New York: Guilford Press.

[eat23228-bib-0018] Fairburn, C. , Norman, P. , Welch, S. , O'Connor, M. , Doll, H. , & Peveler, R. (1995). A prospective study of outcome in bulimia nervosa and the long‐term effects of three psychological treatments. JAMA Psychiatry, 52(4), 304–312. 10.1001/archpsyc.1995.03950160054010 7702447

[eat23228-bib-0019] Fairburn, C. G. (2008). Cognitive behavior therapy and eating disorders. New York: The Guildford Press.

[eat23228-bib-0020] Fairburn, C. G. , Cooper, Z. , Doll, H. , O'Connor, M. E. , Bohn, K. , Hawker, D. , … Palmer, R. (2009). Transdiagnostic cognitive‐behavioral therapy for patients with eating disorders: A two‐site trial with 60‐week follow‐up. American Journal of Psychiatry, 166(3), 311–319. 10.1176/appi.ajp.2008.08040608 19074978 PMC3035831

[eat23228-bib-0021] Fairburn, C. G. , Jones, R. , Peveler, R. C. , Hope, R. A. , & O'Connor, M. (1993). Psychotherapy and bulimia nervosa. Longer‐term effects of interpersonal psychotherapy, behavior therapy, and cognitive behavior therapy. Archives of General Psychiatry, 50(6), 419–428. 10.1001/archpsyc.1993.01820180009001 8498876

[eat23228-bib-0022] Fox, K. R. (1999). The influence of physical activity on mental well‐being. Public Health Nutrition, 2(3a), 411–418. 10.1017/S1368980099000567 10610081

[eat23228-bib-0023] Friborg, O. , Martinussen, M. , Kaiser, S. , Øvergård, K. T. , Martinsen, E. W. , Schmierer, P. , & Rosenvinge, J. H. (2014). Personality disorders in eating disorder not otherwise specified and binge eating disorder: A meta‐analysis of comorbidity studies. The Journal of Nervous and Mental Disease, 202(2), 119–125. 10.1097/nmd.0000000000000080 24469523

[eat23228-bib-0024] Godart, N. T. , Perdereau, F. , Rein, Z. , Berthoz, S. , Wallier, J. , Jeammet, P. , & Flament, M. F. (2007). Comorbidity studies of eating disorders and mood disorders. Critical review of the literature. Journal of Affective Disorders, 97(1), 37–49. 10.1016/j.jad.2006.06.023 16926052

[eat23228-bib-0025] Grant, J. E. , & Chamberlain, S. R. (2019). Neurocognitive findings in young adults with binge eating disorder. International Journal of Psychiatry in Clinical Practice, 14, 1–6. 10.1080/13651501.2019.1687724 PMC7100552 31722589

[eat23228-bib-0026] Graves, T. A. , Tabri, N. , Thompson‐Brenner, H. , Franko, D. L. , Eddy, K. T. , Bourion‐Bedes, S. , … Thomas, J. J. (2017). A meta‐analysis of the relation between therapeutic alliance and treatment outcome in eating disorders. International Journal of Eating Disorders, 50(4), 323–340. 10.1002/eat.22672 28152196

[eat23228-bib-0027] Haugen, T. , Säfvenbom, R. , & Ommundsen, Y. (2011). Physical activity and global self‐worth: The role of physical self‐esteem indices and gender. Mental Health and Physical Activity, 4(2), 49–56. 10.1016/j.mhpa.2011.07.001

[eat23228-bib-0028] Hay, P. (2013). A systematic review of evidence for psychological treatments in eating disorders: 2005–2012. International Journal of Eating Disorders, 46(5), 462–469. 10.1002/eat.22103 23658093

[eat23228-bib-0029] Hedges, G. , & Olkin, I. (1985). Statistical methods in meta‐analysis. Orlando, FL: Academic Press.

[eat23228-bib-0030] Hsu, L. K. G. , Rand, W. , Sullivan, S. , Liu, D. W. , Mulliken, B. , McDonagh, B. , & Kaye, W. H. (2001). Cognitive therapy, nutritional therapy and their combination in the treatment of bulimia nervosa. Psychological Medicine, 31(5), 871–879. 10.1017/S003329170100410X 11459384

[eat23228-bib-0031] Kerrigan, S. G. , Lydecker, J. A. , & Grilo, C. M. (2019). Associations between physical activity and eating‐disorder psychopathology among individuals categorised with binge‐eating disorder and bulimia nervosa. International Journal of Clinical Practice, 73(11), e13401 10.1111/ijcp.13401 31397950 PMC7112656

[eat23228-bib-0032] Keski‐Rahkonen, A. , & Mustelin, L. (2016). Epidemiology of eating disorders in Europe: Prevalence, incidence, comorbidity, course, consequences, and risk factors. Current Opinion in Psychiatry, 29(6), 340–345. 10.1097/yco.0000000000000278 27662598

[eat23228-bib-0033] Laessle, R. G. , Beumont, P. J. V. , Butow, P. , Lennerts, W. , O'Connor, M. , Pirke, K. M. , … Waadt, S. (1991). A comparison of nutritional management with stress management in the treatment of bulimia nervosa. British Journal of Psychiatry, 159(2), 250–261. 10.1192/bjp.159.2.250 1773242

[eat23228-bib-0034] Lambourne, K. , & Tomporowski, P. (2010). The effect of exercise‐induced arousal on cognitive task performance: A meta‐regression analysis. Brain Research, 1341, 12–24. 10.1016/j.brainres.2010.03.091 20381468

[eat23228-bib-0035] Linardon, J. (2018). Rates of abstinence following psychological or behavioral treatments for binge‐eating disorder: Meta‐analysis. International Journal of Eating Disorders, 51(8), 785–797. 10.1002/eat.22897 30058074

[eat23228-bib-0036] Linardon, J. , Fairburn, C. G. , Fitzsimmons‐Craft, E. E. , Wilfley, D. E. , & Brennan, L. (2017). The empirical status of the third‐wave behaviour therapies for the treatment of eating disorders: A systematic review. Clinical Psychology Review, 58, 125–140. 10.1016/j.cpr.2017.10.005 29089145

[eat23228-bib-0037] Linardon, J. , Hindle, A. , & Brennan, L. (2018). Dropout from cognitive‐behavioral therapy for eating disorders: A meta‐analysis of randomized, controlled trials. International Journal of Eating Disorders, 51(5), 381–391. 10.1002/eat.22850 29493805

[eat23228-bib-0038] Linardon, J. , Messer, M. , & Fuller‐Tyszkiewicz, M. (2018). Meta‐analysis of the effects of cognitive‐behavioral therapy for binge‐eating–type disorders on abstinence rates in nonrandomized effectiveness studies: Comparable outcomes to randomized, controlled trials? International Journal of Eating Disorders, 51(12), 1303–1311. 10.1002/eat.22986 30584663

[eat23228-bib-0039] Linardon, J. , & Wade, T. D. (2018). How many individuals achieve symptom abstinence following psychological treatments for bulimia nervosa? A meta‐analytic review. International Journal of Eating Disorders, 51(4), 287–294. 10.1002/eat.22838 29417609

[eat23228-bib-0040] Lowe, C. J. , Hall, P. A. , Vincent, C. M. , & Luu, K. (2014). The effects of acute aerobic activity on cognition and cross‐domain transfer to eating behavior. Frontiers in Human Neuroscience, 8(267), 1–7. 10.3389/fnhum.2014.00267 24808850 PMC4011066

[eat23228-bib-0041] Lubans, D. , Richards, J. , Hillman, C. , Faulkner, G. , Beauchamp, M. , Nilsson, M. , … Biddle, S. (2016). Physical activity for cognitive and mental health in youth: A systematic review of mechanisms. Pediatrics, 138(3), e20161642 10.1542/peds.2016-1642 27542849

[eat23228-bib-0042] Ludwig, K. , & Rauch, W. A. (2018). Associations between physical activity, positive affect, and self‐regulation during preschoolers' everyday lives. Mental Health and Physical Activity, 15, 63–70. 10.1016/j.mhpa.2018.07.002

[eat23228-bib-0043] Martinussen, M. , Friborg, O. , Schmierer, P. , Kaiser, S. , Øvergård, K. T. , Neunhoeffer, A.‐L. , … Rosenvinge, J. H. (2017). The comorbidity of personality disorders in eating disorders: A meta‐analysis. Eating and Weight Disorders—Studies on Anorexia, Bulimia and Obesity, 22(2), 201–209. 10.1007/s40519-016-0345-x 27995489

[eat23228-bib-0044] Mathisen, T. , Rosenvinge, J. H. , Pettersen, G. , Friborg, O. , Vrabel, K. , Bratland‐Sanda, S. , … Sundgot‐Borgen, J. (2017). The PED‐t trial protocol: The effect of physical exercise‐ and dietary therapy compared with cognitive behavior therapy in treatment of bulimia nervosa and binge eating disorder. BMC Psychiatry, 17(1), 180 10.1186/s12888-017-1312-4 28494809 PMC5427572

[eat23228-bib-0045] Mathisen, T. F. , Bratland‐Sanda, S. , Rosenvinge, J. H. , Friborg, O. , Pettersen, G. , Vrabel, K. A. , & Sundgot‐Borgen, J. (2018). Treatment effects on compulsive exercise and physical activity in eating disorders. Journal of Eating Disorders, 6(1), 43 10.1186/s40337-018-0215-1 30559966 PMC6293524

[eat23228-bib-0046] Mathisen, T. F. , Sundgot‐Borgen, J. , Rosenvinge, J. H. , & Bratland‐Sanda, S. (2018). Managing risk of non‐communicable diseases in women with bulimia nervosa or binge eating disorders: A randomized trial with 12 months follow‐up. Nutrients, 10(12), 1887 10.3390/nu10121887 PMC6315508 30513892

[eat23228-bib-0047] Meyer, C. , & Taranis, L. (2011). Exercise in the eating disorders: Terms and definitions. European Eating Disorders Review, 19(3), 169–173. 10.1002/erv.1121 21584910

[eat23228-bib-0048] Mulkens, S. , de Vos, C. , de Graaff, A. , & Waller, G. (2018). To deliver or not to deliver cognitive behavioral therapy for eating disorders: Replication and extension of our understanding of why therapists fail to do what they should do. Behaviour Research and Therapy, 106, 57–63. 10.1016/j.brat.2018.05.004 29763767

[eat23228-bib-0049] Mullen, S. P. , & Hall, P. A. (2015). Editorial: Physical activity, self‐regulation, and executive control across the lifespan. Frontiers in Human Neuroscience, 9(614), 1–3. 10.3389/fnhum.2015.00614 26594162 PMC4635401

[eat23228-bib-0050] Oaten, M. , & Cheng, K. (2006). Longitudinal gains in self‐regulation from regular physical exercise. British Journal of Health Psychology, 11(4), 717–733. 10.1348/135910706X96481 17032494

[eat23228-bib-0051] Pettersen, G. , Sørdal, S. , Rosenvinge, J. H. , Skomakerstuen, T. , Mathisen, T. F. , & Sundgot‐Borgen, J. (2017). How do women with eating disorders experience a new treatment combining guided physical exercise and dietary therapy? An interview study of women participating in a randomised controlled trial at the Norwegian School of Sport Sciences. BMJ Open, 7(12), e018588 10.1136/bmjopen-2017-018588 PMC5778312 29259061

[eat23228-bib-0052] Quesnel, D. A. , Libben, M. , Oelke, D. , Clark, M. N. I. , Willis‐Stewart, S. , & Caperchione, C. M. (2018). Is abstinence really the best option? Exploring the role of exercise in the treatment and management of eating disorders. Eating Disorders, 26(3), 290–310. 10.1080/10640266.2017.1397421 29131718

[eat23228-bib-0053] Rø, Ø. , Reas, D. L. , & Stedal, K. (2015). Eating disorder examination questionnaire (EDE‐Q) in Norwegian adults: Discrimination between female controls and eating disorder patients. European Eating Disorders Review, 23(5), 408–412. 10.1002/erv.2372 26094887

[eat23228-bib-0054] Rosenbaum, S. , Tiedemann, A. , Sherrington, C. , Curtis, J. , & Ward, P. B. (2014). Physical activity interventions for people with mental illness: A systematic review and meta‐analysis. The Journal of Clinical Psychiatry, 75(9), 964–974. 10.4088/JCP.13r08765 24813261

[eat23228-bib-1055] Sheehan, D. V. , Lecrubier, Y. , Sheehan, K. H. , Amorim, P. , Janavs, J. , Weiller, E. et al. (1998). The Mini‐International Neuropsychiatric Interview (M.I.N.I.): the development and validation of a structured diagnostic psychiatric interviewfor DSM‐IV and ICD‐10. J Clin Psychiatry, 59(Suppl 20), 22–33. 9881538

[eat23228-bib-0055] Södersten, P. , Bergh, C. , Leon, M. , Brodin, U. , & Zandian, M. (2017). Cognitive behavior therapy for eating disorders versus normalization of eating behavior. Physiology and Behavior, 174, 178–190. 10.1016/j.physbeh.2017.03.016 28322911

[eat23228-bib-0056] Stubbs, B. , Vancampfort, D. , Hallgren, M. , Firth, J. , Veronese, N. , Solmi, M. , … Kahl, K. G. (2018). EPA guidance on physical activity as a treatment for severe mental illness: A meta‐review of the evidence and position statement from the European psychiatric association (EPA), supported by the International Organization of Physical Therapists in Mental Health (IOPTMH). European Psychiatry, 54, 124–144. 10.1016/j.eurpsy.2018.07.004 30257806

[eat23228-bib-0057] Sundgot‐Borgen, J. , Rosenvinge, J. , Bahr, R. , & Schneider, L. (2002). The effect of exercise, cognitive therapy, and nutritional counseling in treating bulimia nervosa. Medicine & Science in Sports & Exercise, 34(2), 190–195. 11828224 10.1097/00005768-200202000-00002

[eat23228-bib-0058] Swanson, S. A. , Crow, S. J. , Le Grange, D. , Swendsen, J. , & Merikangas, K. R. (2011). Prevalence and correlates of eating disorders in adolescents. Results from the national comorbidity survey replication adolescent supplement. Archives of General Psychiatry, 68(7), 714–723. 10.1001/archgenpsychiatry.2011.22 21383252 PMC5546800

[eat23228-bib-0059] Twisk, J. , de Boer, M. , de Vente, W. , & Heymans, M. (2013). Multiple imputation of missing values was not necessary before performing a longitudinal mixed‐model analysis. Journal of Clinical Epidemiology, 66(9), 1022–1028. 10.1016/j.jclinepi.2013.03.017 23790725

[eat23228-bib-0060] Vancampfort, D. , Probst, M. , Adriaens, A. , Pieters, G. , De Hert, M. , Stubbs, B. , … Vanderlinden, J. (2014). Changes in physical activity, physical fitness, self‐perception and quality of life following a 6‐month physical activity counseling and cognitive behavioral therapy program in outpatients with binge eating disorder. Psychiatry Research, 219(2), 361–366. 10.1016/j.psychres.2014.05.016 24929440

[eat23228-bib-0061] Vancampfort, D. , Vanderlinden, J. , De Hert, M. , Adámkova, M. , Skjaerven, L. H. , Catalán‐Matamoros, D. , … Probst, M. (2013). A systematic review on physical therapy interventions for patients with binge eating disorder. Disability and Rehabilitation, 35(26), 2191–2196. 10.3109/09638288.2013.771707 23594056

[eat23228-bib-0062] Waller, G. , Stringer, H. , & Meyer, C. (2012). What cognitive behavioral techniques do therapists report using when delivering cognitive behavioral therapy for the eating disorders? Journal of Consulting and Clinical Psychology, 80(1), 171–175. 10.1037/a0026559 22141595

[eat23228-bib-0063] Wilson, G. T. , Fairburn, C. C. , Agras, W. S. , Walsh, B. T. , & Kraemer, H. (2002). Cognitive‐behavioral therapy for bulimia nervosa: Time course and mechanisms of change. Journal of Consulting and Clinical Psychology, 70(2), 267–274. 11952185

[eat23228-bib-0064] Witkiewitz, K. , Falk, D. E. , Kranzler, H. R. , Litten, R. Z. , Hallgren, K. A. , O'Malley, S. S. , … Alcohol Clinical Trials Initiative Workgroup . (2014). Methods to analyze treatment effects in the presence of missing data for a continuous heavy drinking outcome measure when participants drop out from treatment in alcohol clinical trials. Alcoholism, Clinical and Experimental Research, 38(11), 2826–2834. 10.1111/acer.12543 PMC4244651 25421518

